# Supplementary material for: Support Vector Machine as a Supervised Learning for the Prioritization of Novel Potential SARS-CoV-2 Main Protease Inhibitors
Source: Int J Mol Sci. 2021 Jul 19;22(14):7714. doi: 10.3390/ijms22147714 (PMC8305792; doi:10.3390/ijms22147714)

Supporting Information

Support Vector Machine as a supervised learning for the prioritization of novel potential SARS-CoV-2 main protease inhibitors

Nedra Mekni [1,2*], Claudia Coronnello [2], Thierry Langer[1], Maria De Rosa [2†] and Ugo Perricone [2*†]

[1] University of Vienna, Department of Pharmaceutical Chemistry, Althanstraße 14 (UZA II), 1090 Vienna, Austria; nmekni@fondazionerimed.com, thierry.langer@univie.ac.at

[2] Fondazione Ri.MED, Drug Discovery Unit, Palermo, Italy; nmekni@fondazionerimed.com, ccoronnello@fondazionerimed.com, mderosa@fondazionerimed.com, uperricone@fondazionerimed.com

* Correspondence: (N.M.) nmekni@fondazionerimed.com; (U.P.) uperricone@fondazionerimed.com;

† These authors contributed equally to this work

Table S1: Permutation feature importance for calculating relative importance scores of the molecular descriptors identified by the RF-RFE-CV method. In column 2 permuted feature importance score is reported with the deviation standard

Selected descriptor	Permuted feature importance
AATS6i	0.044 ± 0.006
ATSC7m	0.047 ± 0.004
VE1_DzZ	0.086 ± 0.009
SpMax2_Bhm	0.070 ± 0.002
SpMax1_Bhv	0.052 ± 0.005
SpMax2_Bhv	0.076 ± 0.006
CrippenLogP	0.074 ± 0.005

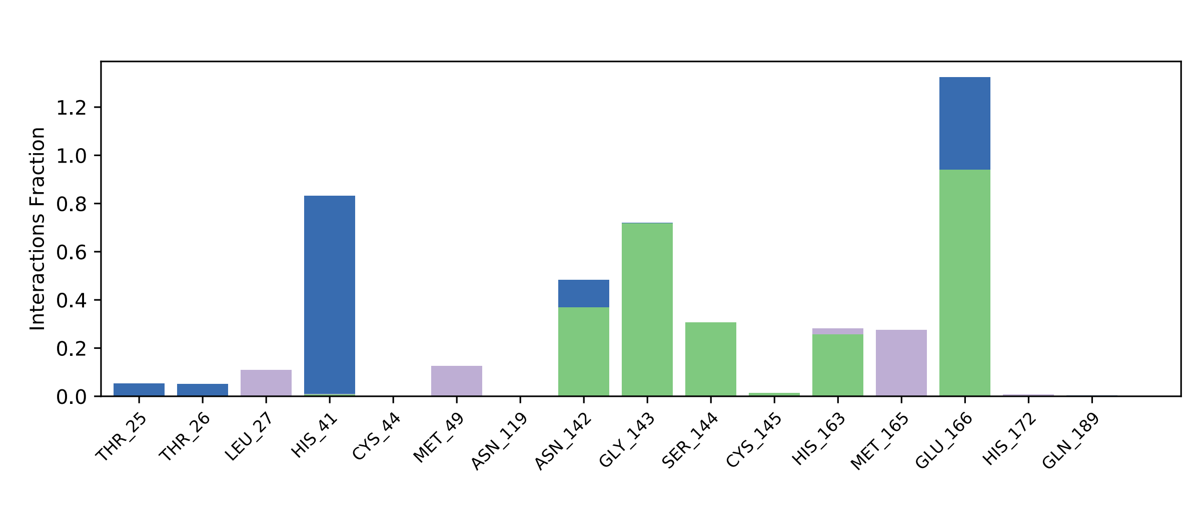

Figure S1: Protein-ligand interaction diagram retrieved from 200ns molecular dynamic simulation performed on PDB 6W63. H-bonds are represented in green, purple hydrophobic interaction and blue interaction mediated by water molecules. In detail Asn 142, Gly 143, Ser 144, His 163, Met 165 and Glu 166 are mainly involved in the protein-ligand stabilization.

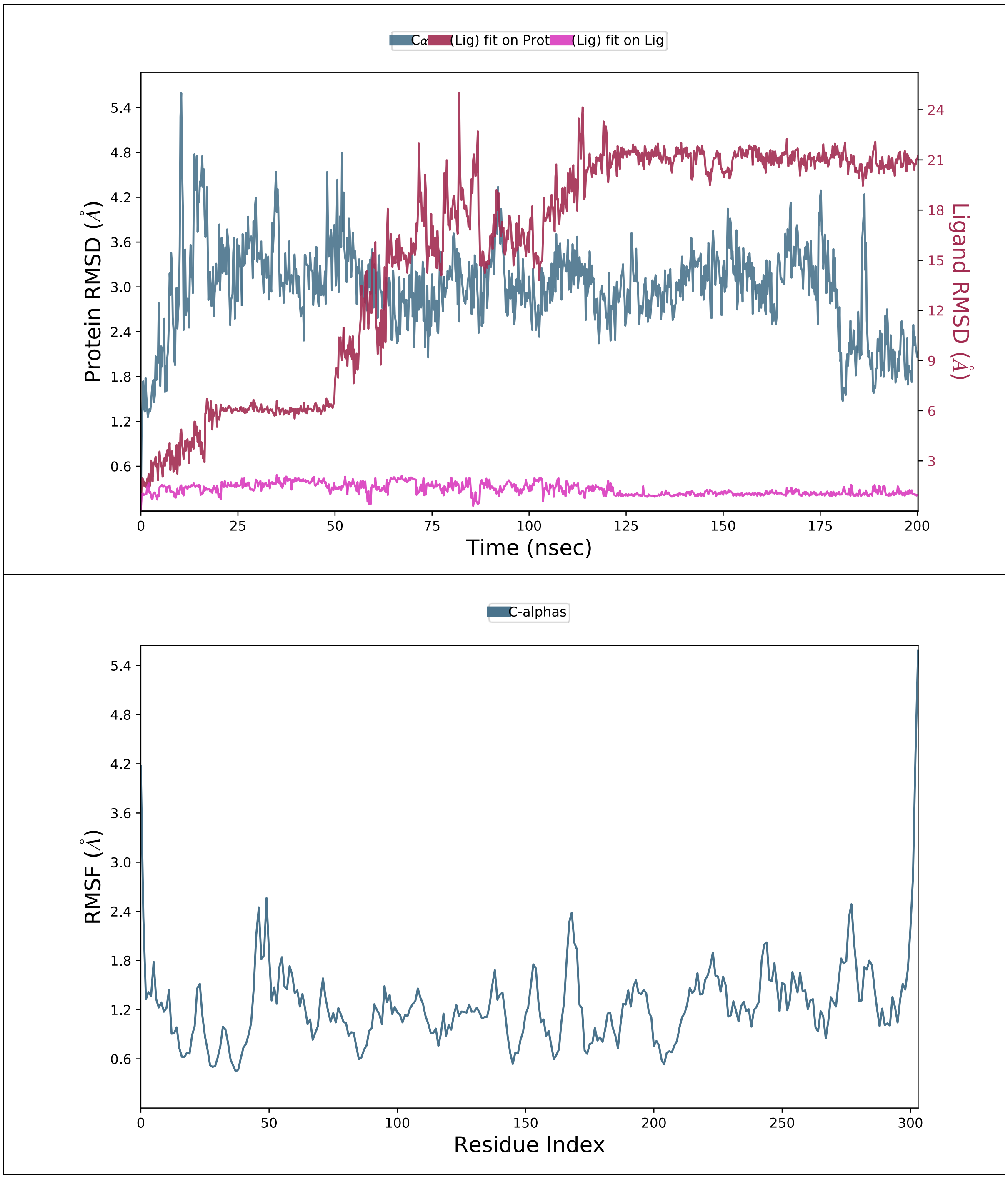

Figure S2: 5RGW molecular dynamic root main square deviation (RMSD) and root mean square fluctuation (RMSF) plots. The plot shows that ligand remained instable during the simulation (200nns).

Table S2: In column 1 are reported the 2D structure of the five consensus compounds retrieved by molecular docking calculation for the evaluation of their binding mode. In column 2 are reported the binding modes on the catalytic site surface.

Compound	2D Structure	Docking pose
I	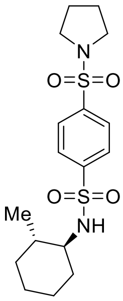	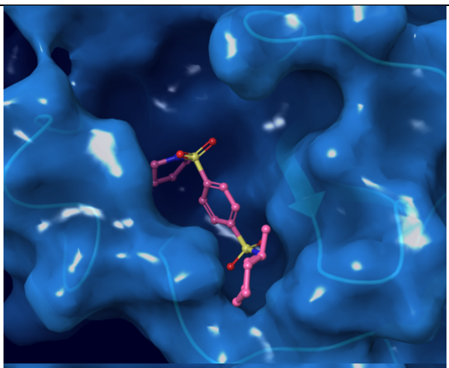
II	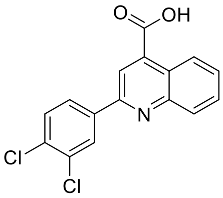	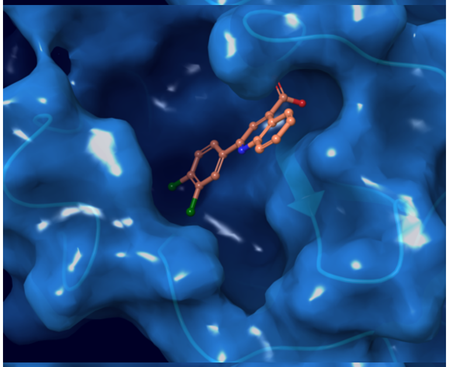
III	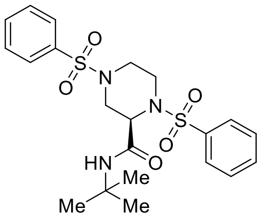	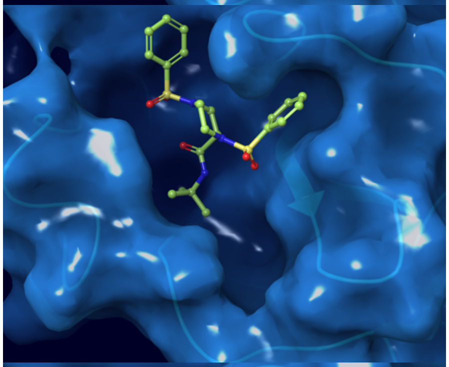
IV	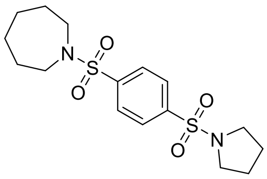	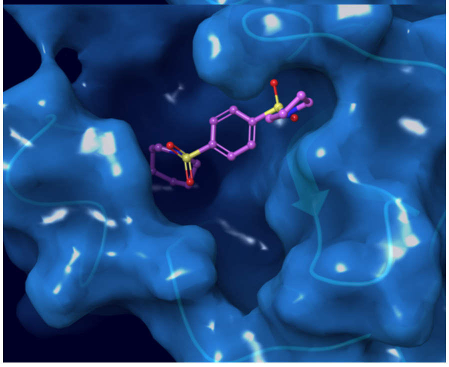

V

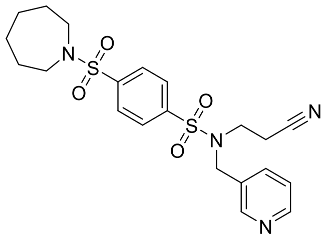

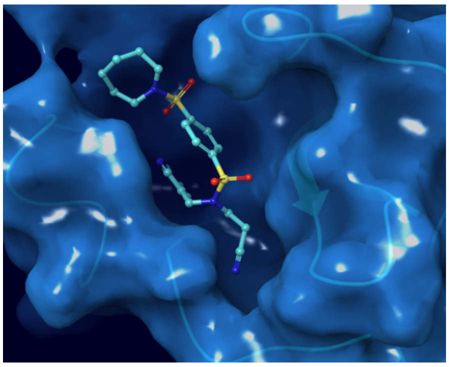

Supplement: Supplementary file 1 [file ijms-22-07714-s001.zip › ijms-1304901-supplementary.pdf]
